# How to Use the New Journalism

**Published:** 1921-02-12

**Authors:** 


### The Hospital

The Workers' Newspaper of Administrative Medicine and Institutional Life, Administration, National Insurance and Health.

No. 1810, Vol. LXIX. SATURDAY, FEBRUARY 12, 1921. PRICE TWOPENCE

### HOW TO USE THE NEW JOURNALISM.

Each hospital is so much immersed in its own affairs that there is little comprehension, outside some study of financial statistics, of the general trend of movement. It is questionable, for instance, how far individual hospitals realise the changes which have come over the practice of publicity, or the effects of the war upon institutional publications generally. Prior to 1914 it may be said, without much fear of contradiction, that, except for hospitals with medical schools, no general hospital published a regular periodical, that asylum journals made practically no appeal to the general public, and that the *Gazettes* did little but record the jokes of medical students, the appointments open to them in their own hospital, and a summary of the best lectures which they heard. If we add to these the modest publications of the great Funds, which in the main were technicalities, garnished by some friendly gossip concerning the staff, there was not much over. We would even ask, in the hope indeed of contradiction, what paper beside The Hospital and the *Hospital Officers' Gazette*, which again was chiefly read by the Association's members, attempted, or even desired, to cover the whole field? Except for the staffs of each of these papers, they were read by very few people outside the immediate circle within their reach, and rarely by each other's readers.

Then came the war. It produced war hospitals like mushrooms. These begat *Gazettes*, and the chief of these, which set a lively pace for its contemporaries, was the outcome of a staff of artists, journalists and penmen. The chief *Gazette* had a large circulation because, at first, the patients in the hospital whose doings it described were a couple of thousand or more. But some of the writers and draughtsmen had already won a following before they entered military hospital life. The result was a paper which might be read with interest by almost anyone. In fact, it was. Then the cost of production increased, nowhere more perhaps than in the printing trade. Advertisements were necessary if the paper was to be able to provide the soldiers with comforts out of its "profits." The circulation made a revenue from advertisements possible. The skill of the staff in the two arts of "black and white" attracted readers. A new standard of achievement was set in institutional journalism; and since success is infectious, the example spread elsewhere.

Now that the war is ended, and the *Gazettes*, like their institutions, have mostly perished, the example has begun to infect pre-war hospitals and papers. The big hospitals without medical schools are beginning to publish their own papers; but the inspiration is now from the hospital secretary, not from the Dean of the medical school. The consequence is that the new hospital paper seeks to interest not only the staff but the neighbouring public, the whole, in fact, of that part of the "greater" city in which it is situated. We see the same thing both in London and in the Provinces. For instance, *Progress*, the journal of the Great Northern Central Hospital, Holloway. Perhaps the latest example is *The G. H. B.*, the organ of the League associated with the General Hospital, Birmingham. We note the same change, again, in the transformation which has come over, and improved, the Metropolitan Hospital Saturday Fund *Journal*. Its change to a monthly review, for that is the goal toward which it is growing, indicates the new importance which hospital journalism has won.

Now all this is a welcome proof of co-operation, for co-operation begins when we cease to be entirely preoccupied with our immediate affairs and try to join hands with those invisible friends on the fringe of our own activities. There is then a new and healthy note of friendly competition. The man who subscribed to all the hospital journals which existed before the war could have spent a pleasant Sunday afternoon instructing himself. The same man today would be possessed of more papers in this one field than he could conveniently read in a whole week. We suggest that this new movement in institutional journalism could go a step further. We invite co-operation between them to the extent of taking in, even of regularly quoting, one another. So far as we are aware, we have reviewed at some length every new paper, and almost every issue of each new paper, which has appeared. We intend to continue this practice. For it is helpful to our readers, and, we trust, not unhelpful to the new recruits. But they would gain a richer harvest still if they made the habit of reviewing and of quoting one another. In this way the whole movement would become linked. One set of readers would attract another, because they would know what each hospital public was reading and doing. Ideas could be interchanged, and the practice and needs of each locality compared, to their mutual advantage. The letters which we received from the editors of the military hospital *Gazettes* showed us that such help as we could give was appreciated. But why should The Hospital be the only common help? Indeed, if we failed to review at once a new paper we were generally reminded of the fact! Notices in advance used to reach us of Special Numbers and so on. We received and returned this advice in a spirit of friendly criticism, and there can be no doubt everyone benefited, including ourselves.

The moral, in fact, is this: Co-operation is still rather an aspiration than a fact. The hospital, no less than the general, public is hardly ripe for it. It is still in the stage of experiment and tentative trial. The case for co-ordination was well argued in the January number of *Progress*, where the closer relation of general and cottage hospitals was advocated. It is noteworthy that this, mainly a provincial problem, was the subject of a leading article in a local London hospital paper. We welcome this broad view. Those, and they are many, who believe that co-operation can be much extended, in finance, in administration, and in the more economic employment of hospital beds must, therefore, create the spirit favourable to a consideration of their plans. An excellent instrument is now to hand in the growing crop of hospital journals. Let not this instrument lie idle! Let all sides meet in institutional journalism, and exchange their readers with their ideas! If any warmth is generated, it will increase the number of both. For the source of energy is heat, and if the hospital journals are warm, it is with the warmth of the fireside and not with that of the fire-eater.

